# Sensitivity and Specificity of Body Mass Index for Sarcopenic Dysphagia Diagnosis among Patients with Dysphagia: A Multi-Center Cross-Sectional Study

**DOI:** 10.3390/nu14214494

**Published:** 2022-10-26

**Authors:** Shintaro Togashi, Hidetaka Wakabayashi, Hironori Ohinata, Shinta Nishioka, Yoji Kokura, Ryo Momosaki

**Affiliations:** 1 Department of Nursing Care, Sendai Kosei Hospital, Sendai 980-0873, Japan; 2 Department of Rehabilitation Medicine, Tokyo Women’s Medical University Hospital, Tokyo 162-8666, Japan; 3 Department of Nursing, International University of Health and Welfare, Chiba 286-8686, Japan; 4 Department of Clinical Nutrition and Food Service, Nagasaki Rehabilitation Hospital, Nagasaki 850-0854, Japan; 5 Department of Nutritional Management, Keiju Hatogaoka Integrated Facility for Medical and Long-Term Care, Hosu 927-0023, Japan; 6 Department of Rehabilitation Medicine, Mie University Graduate School of Medicine, Tsu 514-8507, Japan

**Keywords:** sarcopenia, dysphagia, body mass index, area under curve, cross-sectional study

## Abstract

The accuracy of body mass index (BMI) for sarcopenic dysphagia diagnosis, which remains unknown, was evaluated in this study among patients with dysphagia. We conducted a 19-site cross-sectional study. We registered 467 dysphagic patients aged ≥ 20 years. Sarcopenic dysphagia was assessed using a reliable and validated diagnostic algorithm. BMI was assessed using the area under the curve (AUC) in the receiver operating characteristic analysis to determine diagnostic accuracy for sarcopenic dysphagia. The study included 460 patients (median age, 83.0 years (76.0–88.0); men, 49.8%). The median BMI was 19.9 (17.3–22.6) kg/m^2^. Two hundred eighty-four (61.7%) patients had sarcopenic dysphagia. The AUC for sarcopenic dysphagia was 0.60–0.62 in the overall patients, male, female, and patients aged ≥ 65 years The BMI cut-off value for sarcopenic dysphagia diagnosis was 20.1 kg/m^2^ in the overall patients (sensitivity, 58.1%; specificity, 60.2%) and patients aged ≥ 65 years (sensitivity, 59.8%; specificity, 61.8%). Conclusion: Although the AUC, sensitivity and specificity of BMI for sarcopenic dysphagia diagnosis was approximately 0.6, BMI < 20.0 kg/m^2^ might be a predictor for sarcopenic dysphagia. In clinical settings, if patients with dysphagia have a BMI < 20.0 kg/m^2^, then sarcopenic dysphagia should be suspected as early as possible after admission.

## 1. Introduction

Sarcopenic dysphagia involves swallowing difficulty due to weakening of the skeletal and swallowing muscles, and is common in older people [1,2,3]. The prevalence of sarcopenic dysphagia is approximately 30–40% in hospitalized patients aged ≥ 65 years [4,5]. A single-center prospective cohort study [6] reported that 26% of hospitalized patients, aged ≥ 65 years without dysphagia at admission, without acute stroke, and who had been prohibited oral intake of food for 48 h after admission, developed sarcopenic dysphagia within 60 days after admission. Additionally, several reviews [2,7,8] have reported risk factors for sarcopenic dysphagia to be age, history of clinical disease, lower body mass index (BMI), lower activities of daily living (ADL), malnutrition, skeletal muscle mass loss, lower tongue pressure, and inappropriate nutrition management. Dysphagia negatively affects quality of life and the nationwide medication expense because of aspiration pneumonia, hospital readmission, and longer hospitalization duration [1,7,9]. Therefore, it is important to screen older patients for sarcopenic dysphagia in clinical settings.

BMI is a simple and commonly used measurement to evaluate obesity [10,11] and malnutrition worldwide [12]. Several studies have reported that patients with sarcopenic dysphagia have a significantly lower BMI than those without sarcopenic dysphagia (17.9–18.9 kg/m^2^ vs. 20.3–22.0 kg/m^2^) [3,4,5,6]. Additionally, BMI is associated with the development of dysphagia. Maeda et al. [6] reported a prospective cohort study showing that a low BMI (<20 kg/m^2^) and decreased skeletal muscle mass are independent predictors of newly diagnosed dysphagia within 60 days after admission. Therefore, BMI could be a potentially useful screening method for sarcopenic dysphagia, which is simpler than existing tests and less burdensome for patients.

Currently, the BMI cut-off value and the diagnostic indices for sarcopenic dysphagia diagnosis remain unknown. Nakadate et al. [13] and Ikenaga et al. [14] reported that higher BMI was a significant predictor for the resumption of oral intake in stroke patients admitted to rehabilitation wards. Maeda et al. [15] reported that lower BMI indicated more frequent incidence of swallowing disorders at discharge among older patients without diseases related to swallowing difficulties. Additionally, Kimura et al. [16] reported that calf circumference predicted an improvement in the food intake level scale. However, there are no data regarding BMI cut-off values for identifying sarcopenic dysphagia at the time of admission and the area under the curve (AUC), sensitivity, and specificity of BMI cut-off value for sarcopenic dysphagia at the time of admission. Early identification of sarcopenic dysphagia can prevent adverse events, such as aspiration pneumonia, hospital readmission, longer hospitalization duration, and lower quality of life [14], due to undiagnosed and untreated sarcopenic dysphagia in clinical settings.

Therefore, in this study, we aimed (1) to identify the cut-off values of BMI for sarcopenic dysphagia diagnosis and (2) to determine the AUC, sensitivity, and specificity of BMI for sarcopenic dysphagia diagnosis in patients with dysphagia.

## 2. Materials and Methods

### 2.1. Study Design

We conducted a 19-site cross-sectional study to evaluate the validity of BMI for sarcopenic dysphagia diagnosis among patients with dysphagia. Additionally, we analyzed the receiver operating characteristic (ROC) curve and estimated the BMI cut-off value for sarcopenic dysphagia diagnosis by gender, age ≥ 65 years, and different clinical settings.

The results are reported according to the Standards for Reporting Diagnostic Accuracy (STARD) 2015 statement (Supplementary Table S1) [17]. The study was registered in the University Hospital Medical Information Network Clinical Trial Registry (UMIN-CTR ID: UMIN000038281) and approved by the Institutional Review Board of Yokohama City University Medical Center (Approval No.: B190700074. Approval Date: 7 August 2019.). All participants provided written informed consent before enrollment or were given the right to refuse participation with an opt-out form.

### 2.2. Data Source

Data were derived from a multi-center prospective cohort study using the Japanese Sarcopenic Dysphagia Database, the details of which are described elsewhere [18,19], with a web-based electronic data-capturing system (REDCap) [20]. In the database, we registered dysphagic patients aged ≥ 20 years with a score of ≤8 by the food intake level scale (FILS) [21] at nine acute-care hospitals, eight rehabilitation hospitals, two long-term care hospitals, and one home-visit rehabilitation team between November 2019 and March 2021. FILS scores of 1–3 indicated various degrees of non-oral food intake; 4–6 indicated various degrees of oral food intake and alternative nutrition; 7–8 indicated various degrees of oral food intake alone; 9 indicated no dietary restriction, but with given medical consideration; and 10 indicated normal oral food intake. In this study, we analyzed the baseline data from acute-care, rehabilitation, or long-term care hospitals in the database.

### 2.3. Participants

We included non-consecutive dysphagic in patients aged ≥20 years with a FILS score ≤ 8 [21] between November 2019 and March 2021 in the database. The inclusion criteria was registration in the database [16,18,19,22], while the exclusion criteria were (1) outpatients and (2) missing values for BMI and other components of the diagnostic algorithm for sarcopenic dysphagia, such as muscle strength, physical performance, or muscle mass. The reason for choosing these criteria was that BMI was intended to be used to screen for sarcopenic dysphagia at the time of admission.

### 2.4. Index Test

BMI was utilized as the index test, which was intended to be used for screening. We collected weight and height measurements using a measuring device at baseline or admission. In the case of bedridden or sedentary patients, self-reported data by the patient or a family member were used. BMI was calculated as weight divided by height squared (kg/m^2^). BMI data were handled as both continuous and categorical variables, the latter of which were divided into five categories: <18.5 kg/m^2^ (underweight), 18.5–22.9 kg/m^2^ (low-normal weight), 23.0–24.9 kg/m^2^ (high-normal weight), 25.0–29.9 kg/m^2^ (overweight), and ≥30.0 kg/m^2^ (obese), based on the World Health Organization classification that was modified for the Asian population [3,21] and similar to those used in previous studies [15,23,24,25,26].

### 2.5. Reference Test

The presence of sarcopenic dysphagia was used as the reference test. The index test and reference test were used unblinded. Sarcopenic dysphagia was diagnosed using a reliable and validated diagnostic algorithm [3,27].

First, the presence or absence of sarcopenia was diagnosed using the 2019 criteria of the Asian Working Group for Sarcopenia [3,28]. Muscle mass was evaluated using dual-energy X-ray absorptiometry (DXA), bioimpedance analysis (BIA), or calf circumference (CC). Handgrip strength (HG) was measured to assess muscle strength mainly using the Smedley dynamometer (92.9% of site), followed by the Jamar dynamometer (Model number, MG-4800, Moritoh corporation, Aichi, Japan) (7.1% of site). Physical function was evaluated using usual gait speed or the five-times sit-to-stand test. The diagnostic criteria for sarcopenia [3,28] were low muscle mass (appendicular skeletal muscle mass < 7.0 kg/m^2^ and <5.4 kg/m^2^ by DXA; <7.0 kg/m^2^ and <5.7 kg/m^2^ by BIA; and CC < 34 cm and <33 cm in men and women, respectively) and low muscle strength (HG < 28.0 kg in men and <18.0 kg in women) or low physical performance (five-times sit-to-stand time < 12 s and walking speed < 1.0 m/s).

Subsequently, while the diagnostic algorithm divided the patients into three categories (probable, possible, or no sarcopenic dysphagia), we only divided the patients into two categories (probable/possible or no sarcopenic dysphagia) to evaluate the diagnostic validity of BMI.

### 2.6. Other Variables

We collected the following data according to the standard procedures for data collection: age, gender, primary disease diagnosed using the 10th Revision of the International Statistical Classification of Diseases and Related Health Problems (ICD-10), the Barthel index, malnutrition diagnosed using the Global Leadership Initiative on Malnutrition (GLIM) criteria [17], and other clinical parameters. We published sample data in Supplementary Material as part of a previous study [18].

### 2.7. Statistical Analysis

First, we described the patient characteristics using the descriptive statistics of medians and interquartile range (IQR) for continuous variables and number (%) for categorical variables. Differences between groups were analyzed using the Welch’s *t*-test (parametric) or Mann–Whitney U test (nonparametric), as appropriate. Categorical data are expressed as the frequency (percentage), and differences were analyzed using the Fisher’s exact test (when including any expected value ≤ 5) or Chi-square test (others). Additionally, we described the descriptive statistics of BMI according to the presence and absence of sarcopenic dysphagia, gender, and subgroups of patients without sarcopenic dysphagia (stratified by dysphagia causes). We plotted a histogram of BMI for patients with and without sarcopenic dysphagia.

Second, we calculated the area under the ROC curve (ROC-AUC) as a measurement of the predictive value of the reference test using the pROC package [29]. Gender, age ≥ 65 years, and hospital type were considered the effect modifiers. The ROC curves were developed by plotting sensitivity vs. 1−specificity, and the optimal cut-off point was defined as the closest point to the upper left-hand corner of the graph (high sensitivity and low (1−specificity)) [30]. Confidence intervals (CI) for the AUCs were computed with 2000 stratified bootstrap replicates.

Finally, we calculated the diagnostic indices, such as sensitivity, specificity, positive predictive value (PPV), negative predictive value (NPV), and 95% CIs, of different BMI categories and the cut-off value by the ROC analysis. CIs of these diagnostic indices were obtained using the Clopper and Pearson method. We listed true positives, false positive true negatives, and false negatives using BMI categories and the cut-off value from the ROC analysis.

We performed data-processing and all statistical analyses using RStudio for Mac (version 4.0.5, R Foundation for Statistical Computing, Vienna, Austria) [31]. We included the R code in this study as File S1 in supplemental material. We did not blind between the assessors of the index test and the reference test.

## 3. Results

**Figure 1:**
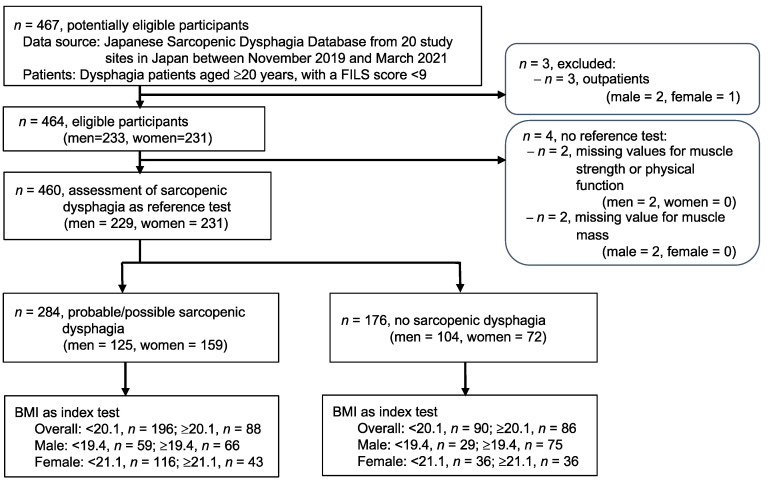
STARD diagram of the patient selection process. Abbreviations: BMI, body mass index; FILS, food intake level scale.

Figure 1 shows the flowchart of the study design. A total of 467 patients had registered in our database. Among the 460 (98.5%) included patients, 229 (49.8%) were men. The median age was 83.0 (IQR, 76.0–88.0) years and the median BMI was 19.9 (IQR, 17.3–22.6) kg/m^2^ (Supplementary Figure S1). There were 284 (61.7%) patients with probable/possible sarcopenic dysphagia. No adverse events were reported from performing the index test or the reference standard.

Table 1 shows the demographic and clinical data of the overall patients and the two subgroups stratified by the presence of sarcopenic dysphagia. The sarcopenic dysphagia group was more likely to include patients who were older, women, and had a BMI < 18.5 kg/m^2^, lower hand grip strength, and malnutrition defined by GLIM. In the sarcopenic dysphagia group, 24 (8.5%) individuals had a BMI ≥ 25 kg/m^2^.

Table 2 shows the descriptive statistics of BMI according to the presence and absence of sarcopenic dysphagia. In case of patients without sarcopenic dysphagia (*n* = 176), we described subgroups according to the cause of dysphagia. Compared to men, there were more women with a BMI < 20 kg/m^2^ and fewer women with no sarcopenic dysphagia. Among 176 participants without sarcopenic dysphagia, the predominant cause of dysphagia was cerebral infarction (78 patients, 44.3%), followed by cerebral hemorrhage (20 patients, 11.4%), Parkinson’s disease (15 patients, 8.5%), dementia (12 patients, 6.8%), cancer (8 patients, 4.5%), and unspecified reasons (33 patients, 18.8%).

**Figure 2:**
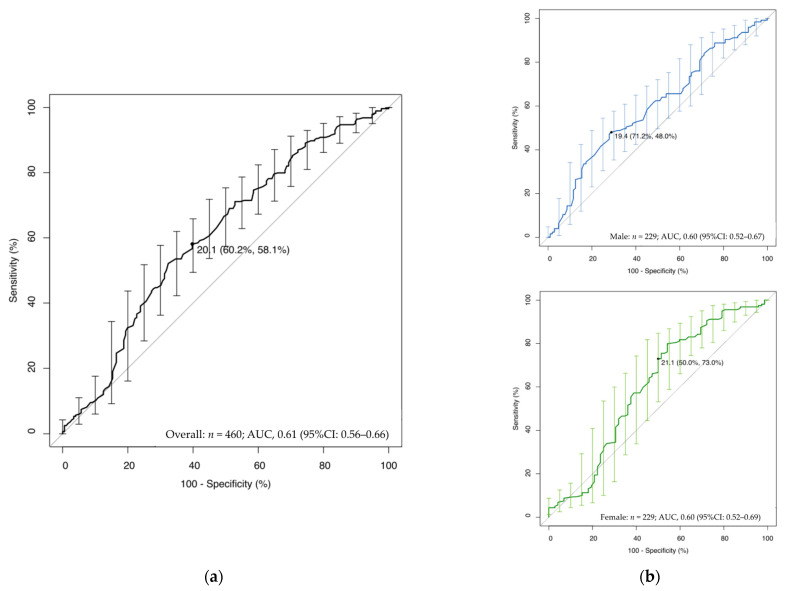
The ROC curve to measure the predictive value of sarcopenic dysphagia. The ROC curve was plotted and the 95% CI of BMI to distinguish between the presence or absence of sarcopenic dysphagia was calculated. Error bar: 95% CI. The CI of the AUC was obtained by 2000 stratified bootstrap replicates. The AUC of the ROC curve was 0.61 (95% CI: 0.56–0.66). (**a**) The optimal BMI cut-off value was 20.1 kg/m^2^ (sensitivity, 58.1% (95% CI: 52.1–63.9); specificity, 60.2% (95% CI: 52.6–67.5)) for the overall patients using the closest top-left method; (**b**) The optimal BMI cut-off values were 19.4 kg/m^2^ in men (sensitivity, 48.0%; specificity, 71.2%), 21.1 kg/m^2^ in women (sensitivity, 73.0%; specificity, 50.0%) using the closest top-left method. Abbreviations: AUC, area under the curve; CI, confidence interval; ROC, receiver operating characteristic.

Figure 2 shows the ROC curve and AUC as measurements of the predictive value of sarcopenic dysphagia. The AUC for sarcopenic dysphagia was 0.61 (95% CI: 0.56–0.66). The BMI cut-off value for sarcopenic dysphagia diagnosis was 20.1 kg/m^2^ in the overall patients (sensitivity, 58.1%; specificity, 60.2%), 19.4 kg/m^2^ in men (sensitivity, 48.0%; specificity, 71.2%), and 21.1 kg/m^2^ in women (sensitivity, 73.0%; specificity, 50.0%).

Table 3 lists the diagnostic indices of BMI (including the AUC, cut-off value, and numbers of true positives, false positives, false negatives, and true negatives) for sarcopenic dysphagia for the overall patient population, patients of both gender, those aged ≥ 65 years, and those from different clinical settings according to the cut-off value defined using the ROC curve and BMI category. The AUC was 0.60 (95% CI: 0.52–0.67) in men, 0.60 (95% CI: 0.52–0.69) in women, and 0.62 (95% CI: 0.57–0.68) in patients aged ≥ 65 years. The optimal BMI cut-off values were 19.4 kg/m^2^ in men (sensitivity, 48.0%; specificity, 71.2%), 21.1 kg/m^2^ in women (sensitivity, 73.0%; specificity, 50.0%), and 20.1 kg/m^2^ in patients aged ≥ 65 years (sensitivity, 59.8%; specificity, 61.8%). According to the World Health Organization’s BMI classification for the Asian population [10], the highest sensitivity (95% CI) was 99.6% (98.1–100) for a BMI < 30 kg/m^2^, followed by 91.5% (87.7–94.5) for a BMI < 25.0 kg/m^2^, 82.0% (77.1–86.3) for a BMI < 23.0 kg/m^2^, and 58.1% (52.1–63.9) for a BMI < 20.1 kg/m^2^.

Supplementary Tables S2–S4 show the sensitivity and specificity for sarcopenic dysphagia diagnosis according to various cut-off values for the overall patients and for men and women.

## 4. Discussion

This multi-center cross-sectional study is the first to demonstrate the cut-off value of BMI, AUC, sensitivity, and specificity of BMI for sarcopenic dysphagia diagnosis in patients with dysphagia. The most important finding was that the BMI cut-off point for sarcopenic dysphagia diagnosis was 20.1 kg/m^2^ in the overall patients, 19.4 kg/m^2^ in men, 21.1 kg/m^2^ in women, and 20.1 kg/m^2^ in patients aged ≥ 65 years. Compared to a previous prospective cohort study [6], our study showed a similar BMI cut-off value and lower diagnostic indices. In an acute-care hospital [26], BMI was reportedly associated with dysphagia diagnosis within 60 days after admission and its diagnostic cut-off value was <20 kg/m^2^ (AUC, 0.73 (0.612–0.85); sensitivity, 76.2%; specificity, 68.9%). Regarding the lower diagnostic indices, such as AUC and sensitivity, in comparison to those from a previous study [28], our participants (those with and without sarcopenic dysphagia) had a similar BMI distribution. Particularly, similar BMI distributions were observed among our two subgroups of men (with sarcopenic dysphagia, 20.0 (16.9–22.4) kg/m^2^; without sarcopenic dysphagia, 20.9 (18.8–23.8) kg/m^2^) (Table 2). A previous pooled analysis, with data from 12 countries and 24 studies [32], reported a mean BMI of 23–24 kg/m^2^ and 24–25 kg/m^2^ in acute-care hospitals and rehabilitation hospitals, respectively. Additionally, a previous Japanese nation-wide administrative database study [25] reported that approximately 39% of the inpatients aged ≥ 65 years had a BMI ≥ 23.0 kg/m^2^. These studies showed a higher proportion of patients with a BMI ≥ 23 kg/m^2^ than ours. Therefore, when considering inpatients in clinical settings, a sensitivity higher than our results might occur owing to sampling bias. Conversely, regarding the BMI cut-off value, our cut-off value was almost consistent with the GLIM consensus [12] for the diagnosis of malnutrition in Asian populations aged ≥ 70 years (low BMI < 20.0 kg/m^2^). Therefore, while it may be useful for clinicians to screen for sarcopenic dysphagia using the same BMI cut-off value of <20.0 kg/m^2^ as early as possible after admission, different cut-off values for evaluating men and women, once their condition stabilizes, might be more desirable. A combination of a BMI < 20.0 kg/m^2^ and a malnutrition diagnosis based on the GLIM criteria might increase sensitivity and specificity. Similar to previous studies [5,6], we found that patients with sarcopenic dysphagia had a higher proportion of malnutrition than those without sarcopenic dysphagia (Table 1). Furthermore, for non-Asian patients, the BMI cut-off values might be higher because of their higher BMI compared with the Asian population [15].

The second important finding in our study is that 8.5% (24/284) of the participants with sarcopenic dysphagia had a BMI ≥ 25.0 kg/m^2^. While our results could not be ruled out in patients with a BMI ≥ 25 kg/m^2^, they could almost be ruled out in those with a BMI ≥ 30 kg/m^2^. Furthermore, lip force and/or tongue pressure is a potential useful screening test for sarcopenic dysphagia and obesity. A previous cross-sectional study [17] reported that lip force (AUC, 0.84–0.88) and tongue pressure (AUC, 0.74–0.78) were highly accurate diagnostic indices for sarcopenic dysphagia among inpatients aged ≥ 65 years in the post-acute phase of illness. This previous study has limited information about diagnostic indices among patients with obesity owing to the lack of subgroup analysis. In our study, tongue pressure could not be measured in many patients because the measuring equipment was not available in some hospitals [18]. Therefore, future studies are required to evaluate the diagnostic potential of a screening method using lip force and/or tongue pressure for patients aged ≥ 65 years with sarcopenic dysphagia and obesity.

Sarcopenic obesity is a clinical and functional condition characterized by the coexistence of obesity, characterized by excess fat mass, and sarcopenia [33]. Obesity can independently lead to loss of muscle mass and function, due to the negative impact of adipose tissue-dependent metabolic derangements, such as oxidative stress, inflammation, and insulin resistance, all of which negatively affect muscle mass [34]. Therefore, some patients with sarcopenic obesity might have dysphagia. Because the prevalence of obesity is increasing, the number of patients with both sarcopenic dysphagia and obesity might also be increasing. A previous nationwide annual survey in Japan [35] reported that obesity prevalence increased from 1973 to 2016 among people aged ≥ 65 years. Particularly, the prevalence of obesity had increased from 12.6% to 30.3% in men and from 23.1% to 24.0% in women. While previous reviews of treatments for sarcopenic dysphagia [2,3] for patients with a BMI < 21 kg/m^2^ are available, treatment strategies for sarcopenic dysphagia and obesity remain unknown. Future studies on treatment for patients with sarcopenic dysphagia and obesity are required.

The interesting finding was the difference in median BMI of 1.0 kg/m^2^ between two groups and that we found no significant differences except for subarachnoid hemorrhage in female patients in this study. Our result was the fewer difference in BMI between two group than previous studies [3,4,5,6]. We hypothesized that patients with cerebrovascular disease might have been affected by a higher BMI, while cancer and dementia might have been affected by a lower BMI, thereby affecting diagnostic accuracy. Contrary to our hypothesis, patients with dysphagia had similar BMI despite the presence of sarcopenia and regardless of the cause of dysphagia, and we found no significant differences except for subarachnoid hemorrhage in women.

### 4.1. Clinical Implications

Our findings have important clinical implications as they suggest that screening for sarcopenic dysphagia using BMI as early as possible after admission may prevent adverse events, such as aspiration pneumonia, readmissions, longer hospitalization, and lower quality of life [31], due to undiagnosed and untreated sarcopenic dysphagia. First, in patients with dysphagia and a BMI < 20.0 kg/m^2^, clinicians should suspect the possibility of sarcopenic dysphagia. Second, in cases of sarcopenic dysphagia, treatment involves both dysphagia rehabilitation, such as resistance training of swallowing muscles, and nutritional support [33]. It is important for clinicians to screen for sarcopenic dysphagia as early as possible after admission using BMI, a simple, non-invasive, and commonly used measurement.

### 4.2. Limitations

This study had some limitations and methodological biases for diagnostic test accuracy [36].

First, we did not consider the prevalence or development of dysphagia because we did not include patients with dysphagia at admission. However, the duration between the admission date and the date of baseline evaluation was similar between patients with and without sarcopenic dysphagia (median: 3 (2–6) days vs. 3 (2–7) days, respectively; *p* = 0.509 using the Wilcoxon rank sum test). Therefore, sarcopenic dysphagia development after hospitalization was not frequent in our results.

Second, a FILS score < 9 was used as a screening test for dysphagia, instead of instrumental swallowing evaluation. In this regard, while recommendations for the use of evaluation tools for dysphagia remain controversial [37], a previous cross-sectional study [38] demonstrated that all patients with a confirmed diagnosis of dysphagia by video fluoroscopic examination of swallowing had a FILS score < 9.

Third, our results might have been influenced by methodological biases such as non-consecutive patient selection causing a sampling bias and no blinding between index test and reference test causing an information bias [36]. Our sampling method was not consecutive and random. Participants with a more advanced age (≥85 years, 44%) and lower Barthel index score (51% total assistance, (0–20)) were more common in our study. Additionally, the frequency of patients without sarcopenic dysphagia, who had a low BMI (20.90 (18.30–24.10) kg/m^2^), was higher in our study than that in previous studies [4,5,6]. Therefore, when considering inpatients in clinical settings, a sensitivity higher than that observed in our results might be obtained.

Finally, there is a significant difference in the physiology of people aged 20 years (young adult) and 60 years (old). Our study, which considered age ≥ 65 years as the effect modifier, showed similar BMI cut-off value between overall patients and patients aged ≥ 65 years. However, the sample number of 36 patients made it difficult to analyze the factors contributing to sarcopenic dysphagia in younger patients. Therefore, future studies to evaluate the prevalence of sarcopenic dysphagia and to identify tests for early detection of sarcopenia and sarcopenic dysphagia among patients aged 20–64 years are required.

## 5. Conclusions

While the AUC indicated a low accuracy of BMI for sarcopenic dysphagia diagnosis in the present study, a BMI of 20.0 kg/m^2^ might represent a better cut-off value among patients with dysphagia. In clinical settings, if patients with dysphagia have a BMI < 20.0 kg/m^2^, then screening for sarcopenic dysphagia should be started as early as possible after admission.

## Figures and Tables

**Table 1:** The demographic and clinical data of patients with and without sarcopenic dysphagia.

Variable	Level	Overall	Sarcopenic Dysphagia		
			Probable/Possible	No	*p* Value
*n*		460	284	176	
Age, years (median (IQR))		83.0 (76.0, 88.0)	85.0 (79.0, 89.0)	78.0 (70.0, 85.0)	<0.001
Age category (%)	<65 years	36 (7.8)	12 (4.2)	24 (13.6)	<0.001
	65–74 years	64 (13.9)	27 (9.5)	37 (21.0)	
	75–84 years	159 (34.6)	92 (32.4)	67 (38.1)	
	≥85 years	201 (43.7)	153 (53.9)	48 (27.3)	
Gender (%)	Male	229 (49.8)	125 (44.0)	104 (59.1)	0.002
	Female	231 (50.2)	159 (56.0)	72 (40.9)	
Hospital type (%)	Acute hospital	202 (43.9)	124 (43.7)	78 (44.3)	0.94
	Rehabilitation hospital	208 (45.2)	130 (45.8)	78 (44.3)	
	Long-term hospital	50 (10.9)	30 (10.6)	20 (11.4)	
FILS at baseline (%)	1	80 (17.4)	28 (9.9)	52 (29.5)	<0.001
	2	34 (7.4)	24 (8.5)	10 (5.7)	
	3	15 (3.3)	11 (3.9)	4 (2.3)	
	4	7 (1.5)	3 (1.1)	4 (2.3)	
	5	9 (2.0)	8 (2.8)	1 (0.6)	
	6	30 (6.5)	22 (7.7)	8 (4.5)	
	7	167 (36.3)	100 (35.2)	67 (38.1)	
	8	118 (25.7)	88 (31.0)	30 (17.0)	
Primary diagnosis that led to hospitalization (%)	Diseases of the circulatory system	165 (35.9)	68 (23.9)	97 (55.1)	<0.001
	Cerebrovascular disease	130 (28.3)	41 (14.4)	89 (50.6)	
	Injury, poisoning, and certain other consequences of external causes	135 (29.3)	105 (37.0)	30 (17.0)	
	Diseases of the respiratory system	57 (12.4)	50 (17.6)	7 (4.0)	
	Diseases of the nervous system	26 (5.7)	10 (3.5)	16 (9.1)	
	Cancer	25 (5.4)	10 (3.5)	15 (8.5)	
	Diseases of the musculoskeletal system and connective tissue	18 (3.9)	16 (5.6)	2 (1.1)	
	Diseases of the digestive system	13 (2.8)	11 (3.9)	2 (1.1)	
	Diseases of the genitourinary system	13 (2.8)	9 (3.2)	4 (2.3)	
	Endocrine, nutritional, and metabolic diseases	4 (0.9)	3 (1.1)	1 (0.6)	
	Diseases of the skin and subcutaneous tissue	2 (0.4)	1 (0.4)	1 (0.6)	
	Infectious disease	1 (0.2)	1 (0.4)	0 (0.0)	
	Missing	1 (0.2)	0 (0.0)	1 (0.6)	
BMI, kg/m^2^ (median (IQR))		19.9 (17.3, 22.6)	19.4 (17.0, 21.9)	20.9 (18.3, 24.1)	<0.001
BMI category (%)	<18.5 kg/m^2^	171 (37.2)	123 (43.3)	48 (27.3)	0.001
	18.5–22.9 kg/m^2^	184 (40.0)	110 (38.7)	74 (42.0)	
	23.0–24.9 kg/m^2^	50 (10.9)	27 (9.5)	23 (13.1)	
	25.0–29.9 kg/m^2^	50 (10.9)	23 (8.1)	27 (15.3)	
	≥30.0 kg/m^2^	5 (1.1)	1 (0.4)	4 (2.3)	
Barthel index score (median (IQR))		20.0 (5.0, 50.0)	20.0 (0.0, 50.0)	22.50 (10.0, 50.0)	0.320
Barthel index (%)	Independent (100)	5 (1.1)	1 (0.4)	4 (2.3)	0.155
	Partial assistance (21–99)	222 (48.3)	138 (48.6)	84 (47.7)	
	Total assistance (0–20)	233 (50.7)	145 (51.1)	88 (50.0)	
Calf circumference, cm (median (IQR))		28.0 (25.1, 31.0)	27.0 (24.5, 29.5)	30.5 (27.1, 32.5)	<0.001
Hand grip strength, kg (median (IQR))		12.0 (6.3, 18.7)	10.8 (6.0, 15.0)	16.7 (7.1, 25.0)	<0.001
GLIM malnutrition (%)		301 (65.4)	208 (73.2)	93 (52.8)	<0.001
Duration between admission and evaluation, days (median (IQR))		3.0 (2.0, 6.0)	3.0 (2.0, 7.0)	3.0 (2.0, 6.0)	0.509

Abbreviations: BMI, body mass index; CCI, Charlson comorbidity index; FILS, food intake level scale; GLIM, Global Leadership Initiative on Malnutrition; IQR, interquartile range.

**Table 2:** The descriptive statistics of BMI according to the presence or absence of sarcopenic dysphagia. In cases of patients without sarcopenic dysphagia (*n* = 176), we describe subgroups according to the cause of dysphagia.

Group		*n*	BMI < 20 kg/m^2^ (%)	Min (kg/m^2^)	Q25 (kg/m^2^)	Median (kg/m^2^)	Q75 (kg/m^2^)	Max (kg/m^2^)
Overall		460	50.2	9.9	17.3	19.9	22.6	32.3
Probable/possible sarcopenic dysphagia		284	56.7	9.9	17.0	19.4	21.9	32.3
-Men		125	49.6	13.8	16.9	20.0	22.4	32.3
-Women		159	62.3	9.9	17.0	19.2	21.2	29.9
No sarcopenic dysphagia		176	39.8	12.2	18.3	20.9	24.1	30.8
Subgroup of no sarcopenic dysphagia according to gender and cause of dysphagia								
Gender	Cause of dysphagia	*n*	BMI < 20 kg/m^2^ (%)	Min (kg/m^2^)	Q25 (kg/m^2^)	Median (kg/m^2^)	Q75 (kg/m^2^)	Max (kg/m^2^)
Men		104	35.6	12.2	18.8	20.9	23.8	30.8
-Subgroup	Cerebral infarction	60	26.7	12.2	19.6	21.6	24.3	30.5
	Cerebral hemorrhage	10	40.0	16.2	18.4	22.2	26.1	30.8
	Subarachnoid hemorrhage	3	33.3	14.9	17.7	20.5	22.4	24.2
	Parkinson’s disease	9	55.6	15.4	18.0	19.9	21.1	26.4
	Dementia	1	0	20.1	20.1	20.1	20.1	20.1
	Cancer	7	71.4	14.3	15.8	17.0	19.8	20.1
	Unspecified reasons	13	46.2	15.7	18.9	20.3	22.3	24.7
	Missing	1	0	24.2	24.2	24.2	24.2	24.2
Gender	Cause of dysphagia	*n*	BMI < 20 kg/m^2^ (%)	Min (kg/m^2^)	Q25 (kg/m^2^)	Median (kg/m^2^)	Q75 (kg/m^2^)	Max (kg/m^2^)
Women		72	45.8	13.5	17.4	20.8	24.5	30.2
-Subgroup	Cerebral infarction	18	44.4	14.2	16.1	21.6	25.9	30.2
	Cerebral hemorrhage	10	40.0	13.5	18.0	20.6	21.8	24.7
	Subarachnoid hemorrhage	6	33.3	14.8	20.6	24.0	26.1	27.2
	Parkinson’s disease	6	83.3	15.6	15.8	16.6	19.2	23.9
	Dementia	11	54.5	13.8	17.1	19.5	22.1	25.8
	Cancer	1	0	22.9	22.9	22.9	22.9	22.9
	Unspecified reasons	20	40.0	14.8	18.8	21.9	25.1	28.4

Abbreviations: BMI, body mass index; Q25, first quartile; Q75, third quartile.

**Table 3:** The diagnostic indices of BMI for sarcopenic dysphagia according to cut-off value by ROC and BMI category for overall patients, patients of both gender, patients aged ≥ 65 years, and different hospital settings.

Variable	AUC (95% CI)	BMI Cut-Off,	*n*	Tp (*n*)	Fp (*n*)	Fn (*n*)	Tn (*n*)	Sensitivity	Specificity	PPV	NPV
		kg/m^2^						(95% CI) %	(95% CI) %	(95% CI) %	(95% CI) %
Cut-off as per ROC											
Overall	0.61 (0.56–0.66)	20.1	460	165	70	119	106	58.1 (52.1–63.9)	60.2 (52.6–67.5)	70.2 (63.9–76.0)	47.1 (40.4–53.9)
Subgroup											
Men	0.60 (0.52–0.67)	19.4	229	59	29	66	75	47.2 (38.2–56.3)	72.1 (62.5–80.5)	67.0 (56.2–76.7)	53.2 (44.6–61.6)
Women	0.60 (0.52–0.69)	21.1	231	116	36	43	36	73.0 (65.3–79.7)	50.0 (38.0–62.0)	76.3 (68.7–82.8)	45.6 (34.3–57.2)
Patients aged ≥ 65 years	0.62 (0.57–0.68)	20.1	424	163	58	109	94	59.8 (53.8–65.8)	61.8 (53.6–69.6)	73.8 (67.4–79.4)	46.3 (39.3–53.4)
Acute hospital	0.62 (0.54–0.70)	19.4	202	61	24	63	54	49.2 (40.1–58.3)	69.2 (57.8–79.2)	71.8 (61.0–81.0)	46.2 (36.9–55.6)
Rehabilitation hospital	0.59 (0.51–0.68)	19.6	208	71	28	59	50	54.6 (45.7–63.4)	64.1 (52.4–74.7)	71.7 (61.8–80.3)	45.9 (36.3–55.7)
Long-term care hospital	0.60 (0.44–0.76)	19.9	50	16	6	14	14	53.3 (34.3–71.7)	70.0 (45.7–88.1)	57.1 (37.2–75.5)	63.6 (40.6–82.8)
Cut-off based on the WHO BMI classification											
Overall		18.5	460	123	48	161	128	43.3 (37.5–49.3)	72.7 (65.5–79.2)	71.9 (65.6–78.5)	44.3 (49.8–61.5)
		23	460	233	122	51	54	82.0 (77.1–86.3)	30.7 (24.0–38.1)	65.6 (60.4–70.6)	51.4 (41.5–61.3)
		25	460	260	145	24	31	91.5 (87.7–94.5)	17.6 (12.3–24.1)	64.2 (59.3–68.9)	56.4 (42.3–69.7)
		30	460	283	172	1	4	99.6 (98.1–100)	2.3 (0.6–5.7)	62.2 (57.6–66.7)	80.0 (28.4–99.5)

CI of AUC was obtained by 2000 stratified bootstrap replicates. CI of diagnostic indices was obtained using the Clopper and Pearson method. Abbreviations: AUC, area under the curve; Tp, true positive; Fp, false positive; Fn, false negative; Tn, true negative; PPV, positive predictive value; NPV, negative predict value; 95% CI, confidence interval.

## Data Availability

The datasets generated and analyzed during the current study are not publicly available due to a license agreement with the Japanese Association of Rehabilitation Nutrition. We had published a sample dataset in the Supplementary Materials as part of a previous study by Mizuno et al.

## Supplementary Materials

The following supporting information can be downloaded at: https://www.mdpi.com/article/10.3390/nu14214494/s1, Figure S1: The histogram of BMI in patients with and without sarcopenic dysphagia; Table S1: STARD 2015 checklist; Table S2: Sensitivity and specificity for sarcopenic dysphagia for overall patients; Table S3: Sensitivity and specificity for sarcopenic dysphagia for male; Table S4: Sensitivity and specificity for sarcopenic dysphagia for female, File S1: Software Source Code.
