# Supplementary material for: Sensitivity and Specificity of Body Mass Index for Sarcopenic Dysphagia Diagnosis among Patients with Dysphagia: A Multi-Center Cross-Sectional Study
Source: Nutrients. 2022 Oct 26;14(21):4494. doi: 10.3390/nu14214494 (PMC9655070; doi:10.3390/nu14214494)
Supplement: Supplementary file 1 [file nutrients-14-04494-s001.zip › nutrients-1967916-supplementary.pdf]

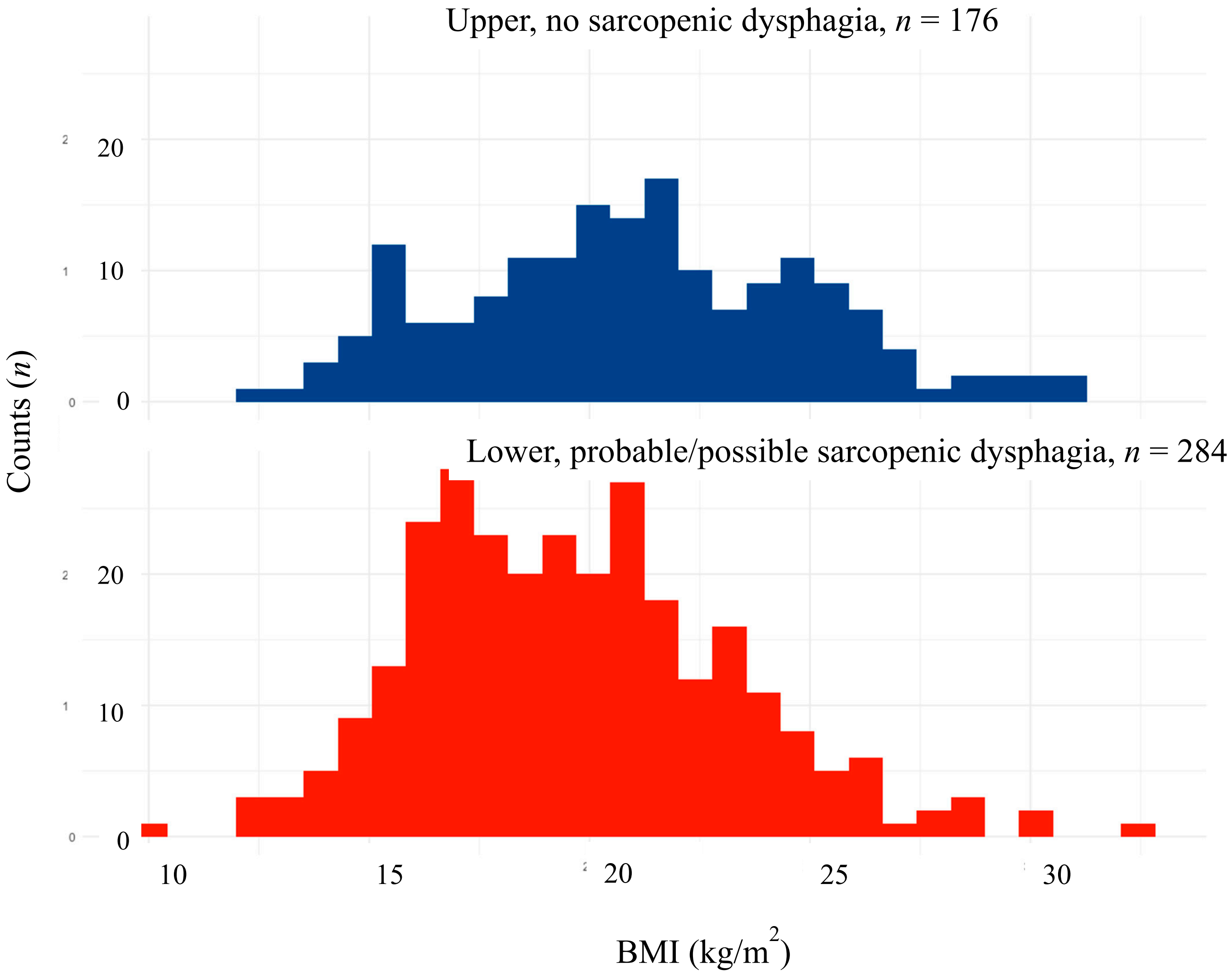

Supplementary Figure S1. The histogram of BMI in patients with and without sarcopenic dysphagia.

Supplementary Table S1. STARD 2015 checklist.

Section & Topic	No	Item	Reported on Page No.
**TITLE OR ABSTRACT**			
	**1**	Identification as a study of diagnostic accuracy using at least one measure of accuracy (such as sensitivity, specificity, predictive values, or AUC)	P1
**ABSTRACT**			
	**2**	Structured summary of study design, methods, results, and conclusions (for specific guidance, see STARD for Abstracts)	P1
**INTRODUCTIO N**			
	**3**	Scientific and clinical background, including the intended use and clinical role of the index test	P1–2
	**4**	Study objectives and hypotheses	P2
**METHODS**			
Study design	**5**	Whether data collection was planned before the index test and reference standard were performed (prospective study) or after (retrospective study)	P2
Participants	**6**	Eligibility criteria	p3
	**7**	On what basis potentially eligible participants were identified (such as symptoms, results from previous tests, inclusion in registry)	P2–3
	**8**	Where and when potentially eligible participants were identified (setting, location and dates)	P2–3
	**9**	Whether participants formed a consecutive, random or convenience series	P3, 11
Test methods	**10a**	Index test, in sufficient detail to allow replication	P3
	**10b**	Reference standard, in sufficient detail to allow replication	P3
	**11**	Rationale for choosing the reference standard (if alternatives exist)	P3
	**12a**	Definition of and rationale for test positivity cut-offs or result categories of the index test, distinguishing pre-specified from exploratory	-
	**12b**	Definition of and rationale for test positivity cut-offs or result categories of the reference standard, distinguishing pre-specified from exploratory	-
	**13a**	Whether clinical information and reference standard results were available to the performers/readers of the index test	P4
	**13b**	Whether clinical information and index test results were available to the assessors of the reference standard	P4
Analysis	**14**	Methods for estimating or comparing measures of diagnostic accuracy	P4
	**15**	How indeterminate index test or reference standard results were handled	P4
	**16**	How missing data on the index test and reference standard were handled	P4
	**17**	Any analyses of variability in diagnostic accuracy, distinguishing pre-specified from exploratory	P4
	**18**	Intended sample size and how it was determined	-
**RESULTS**			
Participants	**19**	Flow of participants, using a diagram	P4, Figure1
	**20**	Baseline demographic and clinical characteristics of participants	P4, Table1
	**21a**	Distribution of severity of disease in those with the target condition	Table1, Figure S1
	**21b**	Distribution of alternative diagnoses in those without the target condition	-

	**22**	Time interval and any clinical interventions between index test and reference standard	Table1
Test results	**23**	Cross tabulation of the index test results (or their distribution) by the results of the reference standard	Table3
	**24**	Estimates of diagnostic accuracy and their precision (such as 95% confidence intervals)	Table3, Figure2
	**25**	Any adverse events from performing the index test or the reference standard	P4
**DISCUSSION**			
	**26**	Study limitations, including sources of potential bias, statistical uncertainty, and generalisability	P11
	**27**	Implications for practice, including the intended use and clinical role of the index test	P10
**OTHER INFORMATION**			
	**28**	Registration number and name of registry	P2, P12
	**29**	Where the full study protocol can be accessed	-
	**30**	Sources of funding and other support; role of funders	P11

Supplementary Table S2: Sensitivity and specificity for sarcopenic dysphagia for overall.

BMI (kg/m²)	Sensitivity	95%CI			Specificity	95%CI		
30.05	99.6	98.9	-	100.0	2.3	0.6	-	4.5
28.75	98.9	97.5	-	100.0	4.0	1.7	-	6.8
28.3	98.2	96.5	-	99.6	4.5	1.7	-	8.0
28.05	97.9	96.1	-	99.3	5.1	2.3	-	8.5
26.5	96.8	94.4	-	98.6	8.0	4.0	-	12.5
26.25	96.5	94.0	-	98.2	9.7	5.7	-	14.2
26.15	95.8	93.3	-	97.9	10.2	5.7	-	14.8
25.55	94.7	91.9	-	97.2	14.8	9.7	-	20.5
25.45	94.0	91.2	-	96.5	15.9	10.8	-	21.6
25.25	93.0	89.8	-	95.8	16.5	11.4	-	22.2
24.95	91.5	88.4	-	94.7	17.6	11.9	-	23.9
24.85	91.2	87.7	-	94.4	18.2	12.5	-	24.4
24.65	90.8	87.7	-	94.0	21.0	15.3	-	27.3
24.45	90.5	87.0	-	93.7	22.2	15.9	-	28.4
24.35	90.1	86.6	-	93.3	22.7	16.5	-	29.0
24.15	89.8	86.3	-	93.0	23.9	17.6	-	30.7
24.05	89.1	85.2	-	92.3	25.6	18.8	-	32.4
23.8	87.3	83.5	-	91.2	26.7	19.9	-	33.5
23.65	87.0	83.1	-	90.8	27.8	21.0	-	34.7
23.45	85.9	82.0	-	90.1	28.4	21.6	-	35.2
23.35	85.2	81.0	-	89.1	29.5	22.7	-	36.4
23.15	84.2	79.6	-	88.4	30.1	23.3	-	36.9
23.05	83.1	78.5	-	87.3	30.7	23.9	-	37.5
22.85	82.0	77.5	-	86.3	31.3	23.9	-	38.1
22.75	80.6	76.1	-	85.2	31.8	25.0	-	39.2
22.55	79.9	75.4	-	84.5	34.1	26.7	-	41.5
22.45	79.6	75.0	-	84.2	35.2	27.8	-	42.0
22.25	78.2	73.2	-	83.1	36.9	29.5	-	44.3
22.15	77.5	72.5	-	82.4	37.5	30.1	-	44.9
21.95	75.7	70.8	-	80.6	38.6	31.3	-	46.0
21.85	74.6	69.7	-	79.6	41.5	34.1	-	48.9
21.55	71.5	66.2	-	76.4	44.3	36.9	-	51.7
21.35	71.1	65.8	-	76.1	47.2	39.8	-	54.5
21.05	69.0	63.7	-	74.3	48.9	41.5	-	56.3
20.95	67.3	62.0	-	72.5	49.4	42.0	-	56.8
20.85	66.5	60.9	-	71.8	50.6	43.2	-	58.0
20.7	65.5	59.9	-	70.8	51.1	43.8	-	58.5
20.55	63.0	57.4	-	68.7	52.8	45.5	-	59.7
20.45	60.6	54.9	-	66.2	55.1	47.7	-	62.5
20.35	59.5	53.9	-	65.1	56.8	49.4	-	63.7
20.25	59.2	53.5	-	64.8	58.0	51.1	-	64.8
20.15	58.5	52.8	-	64.1	58.5	51.1	-	65.9
20.05	58.1	52.5	-	63.7	60.2	52.8	-	67.0
19.85	56.0	50.0	-	61.6	61.4	54.0	-	68.2
19.75	54.9	48.9	-	60.6	63.1	55.7	-	69.9
19.55	53.5	47.5	-	59.2	65.3	58.5	-	72.2
19.45	52.1	46.1	-	57.7	67.6	60.8	-	74.4

19.35	50.0	44.0	-	55.3	68.2	61.4	-	75.0
19.25	48.9	43.0	-	54.2	68.8	61.9	-	75.0
19.05	46.8	40.8	-	52.1	69.3	62.5	-	75.6
18.95	45.4	39.8	-	51.1	69.9	63.1	-	76.7
18.75	44.7	39.1	-	50.4	71.6	64.8	-	77.8
18.65	44.4	38.4	-	50.0	72.2	65.3	-	78.4
18.55	43.7	38.0	-	49.3	72.7	65.9	-	79.0
18.35	40.5	34.9	-	46.1	74.4	68.2	-	80.7
18.25	39.1	33.8	-	44.7	76.1	69.3	-	81.8
17.95	36.6	31.3	-	41.9	77.3	71.0	-	83.0
17.75	35.2	29.9	-	40.5	77.8	71.6	-	83.5
17.65	33.1	28.2	-	38.4	78.4	72.2	-	84.1
17.55	32.4	27.5	-	37.7	80.1	73.9	-	85.8
17.45	31.3	26.1	-	36.6	80.7	74.4	-	86.4
17.25	28.5	23.6	-	33.5	81.3	75.0	-	86.9
16.95	24.6	20.1	-	29.6	83.5	77.8	-	88.6
16.65	20.4	15.8	-	25.0	84.1	78.4	-	89.2
16.55	19.0	14.4	-	23.6	84.7	79.5	-	89.8
16.25	14.4	10.6	-	18.3	85.8	80.7	-	90.9
16.15	14.1	10.2	-	18.0	86.4	81.3	-	90.9
16.05	13.7	9.9	-	17.6	86.9	81.8	-	91.5
15.95	13.0	9.5	-	16.9	87.5	82.4	-	92.0
15.75	10.9	7.7	-	14.4	89.2	84.7	-	93.8
15.65	10.2	7.0	-	13.7	89.8	85.2	-	93.8
15.45	9.5	6.3	-	12.7	91.5	87.5	-	95.5
15.25	8.1	4.9	-	11.3	92.6	88.6	-	96.0
15.05	7.4	4.6	-	10.2	94.3	90.9	-	97.7
14.85	6.0	3.5	-	8.8	94.9	91.5	-	97.7
14.75	5.6	3.2	-	8.5	96.0	93.2	-	98.9
14.55	5.3	2.8	-	7.7	96.6	93.8	-	98.9
14.25	4.2	2.1	-	6.7	97.2	94.3	-	99.4
14.15	3.5	1.8	-	5.6	97.7	95.5	-	99.4
13.95	3.2	1.4	-	5.3	98.3	96.0	-	100.0
13.45	2.5	0.7	-	4.2	99.4	98.3	-	100.0
12.15	0.7	0.0	-	1.8	100.0	100.0	-	100.0

CI were obtained by 2000 stratified bootstrap. Abbreviations; CI, confidence interval.

Supplementary Table S3: Sensitivity and specificity for sarcopenic dysphagia for male.

BMI (kg/m²)	Sensitivity	95%CI			Specificity	95%CI		
30.15	99.2	97.6	-	100.0	2.9	0.0	-	6.7
28.4	98.4	96.0	-	100.0	5.8	1.9	-	10.6
26.6	96.8	93.6	-	99.2	6.7	2.9	-	11.5
26.25	96.0	92.0	-	99.2	8.7	3.8	-	14.4
25.55	93.6	88.8	-	97.6	11.5	5.8	-	18.3
25.45	92.8	88.0	-	96.8	12.5	6.7	-	19.2
25.25	92.0	87.2	-	96.8	13.5	7.7	-	20.2
24.9	91.2	86.4	-	96.0	15.4	8.7	-	23.1
24.65	90.4	84.8	-	95.2	19.2	12.5	-	26.9
24.05	88.8	83.2	-	93.6	24.0	16.3	-	32.7
23.6	86.4	80.0	-	92.0	26.0	18.2	-	34.6
23.45	85.6	79.2	-	91.2	26.9	18.3	-	35.6
23.35	84.0	76.8	-	89.6	28.8	20.2	-	37.5
23.15	82.4	75.2	-	88.8	29.8	21.2	-	38.5
23.05	80.8	73.6	-	87.2	30.8	22.1	-	39.4
22.55	76.0	68.0	-	83.2	32.7	24.0	-	41.3
22.45	75.2	68.0	-	82.4	34.6	26.0	-	43.3
22.25	73.6	65.6	-	80.8	35.6	26.9	-	44.2
21.95	69.6	61.6	-	76.8	36.5	27.9	-	45.2
21.85	68.0	59.2	-	76.0	38.5	29.8	-	48.1
21.35	65.6	57.6	-	73.6	46.2	36.5	-	55.8
21.05	64.0	55.2	-	72.0	48.1	39.4	-	57.7
20.85	62.4	53.6	-	70.4	51.0	41.3	-	60.6
20.7	61.6	52.8	-	69.6	51.9	42.3	-	61.5
20.55	58.4	49.6	-	66.4	54.8	46.1	-	64.4
20.45	53.6	44.8	-	62.4	56.7	47.1	-	66.3
20.35	52.8	44.0	-	61.6	59.6	50.0	-	68.3
20.25	52.0	44.0	-	60.8	61.5	52.9	-	70.2
20.05	50.4	41.6	-	59.2	64.4	54.8	-	73.1
19.85	49.6	40.8	-	58.4	65.4	56.7	-	74.0
19.55	48.8	40.0	-	57.6	68.3	59.6	-	76.9
19.45	48.0	39.2	-	56.8	71.2	62.5	-	79.8
19.35	47.2	38.4	-	56.0	72.1	63.5	-	80.8
19.05	44.0	35.2	-	52.8	73.1	64.4	-	80.8
18.95	43.2	34.4	-	52.0	74.0	65.4	-	81.7
18.8	42.4	33.6	-	51.2	75.0	66.3	-	82.7
18.55	41.6	33.6	-	50.4	76.0	67.3	-	83.7
18.35	37.6	29.6	-	46.4	78.8	70.2	-	85.6
18.15	36.0	28.0	-	44.0	80.8	73.1	-	87.5
17.95	34.4	26.4	-	43.2	82.7	75.0	-	89.4
17.75	33.6	25.6	-	42.4	83.7	76.0	-	90.4
17.65	31.2	23.2	-	39.2	84.6	76.9	-	91.3
16.95	26.4	18.4	-	34.4	87.5	80.8	-	93.3
16.65	21.6	14.4	-	28.8	88.5	81.7	-	94.2
15.95	14.4	8.8	-	20.8	91.3	85.6	-	96.2
15.65	10.4	5.6	-	16.0	93.3	88.5	-	97.1
15.25	6.4	2.4	-	11.2	95.2	90.4	-	99.0

14.55	4.0	0.8	-	8.0	97.1	93.3	-	100.0
14.25	2.4	0.0	-	5.6	98.1	95.2	-	100.0
13.95	1.6	0.0	-	4.0	99.0	97.1	-	100.0

CI were obtained by 2000 stratified bootstrap. Abbreviations; CI, confidence interval.

Supplementary Table S4: Sensitivity and specificity for sarcopenic dysphagia for female

BMI (kg/m²)	Sensitivity	95%CI			Specificity	95%CI		
30.05	100.0	100.0	-	100.0	1.4	0.0	-	4.2
28.3	98.1	95.6	-	100.0	2.8	0.0	-	6.9
27.7	97.5	95.0	-	100.0	4.2	0.0	-	9.7
26.15	96.9	93.7	-	99.4	12.5	5.6	-	20.8
25.65	95.6	92.5	-	98.7	19.4	11.1	-	29.2
25.45	95.0	91.2	-	98.1	20.8	12.5	-	30.6
24.45	91.2	86.8	-	95.0	26.4	16.7	-	36.1
24.25	90.6	86.2	-	95.0	27.8	18.1	-	37.5
23.8	88.1	83.0	-	93.1	29.2	19.4	-	40.3
23.65	87.4	82.4	-	92.5	30.6	20.8	-	41.7
22.85	84.3	78.0	-	89.9	31.9	20.8	-	43.1
22.5	83.0	76.7	-	88.7	36.1	25.0	-	47.2
22.15	81.8	75.5	-	87.4	40.3	29.2	-	51.4
21.95	80.5	74.2	-	86.8	41.7	30.6	-	52.8
21.85	79.9	73.6	-	86.2	45.8	34.7	-	56.9
21.35	75.5	68.6	-	81.8	48.6	37.5	-	59.7
21.05	73.0	66.0	-	79.9	50.0	38.9	-	61.1
20.45	66.0	58.5	-	73.0	52.8	41.7	-	63.9
20.1	64.2	57.2	-	71.7	54.2	43.1	-	65.3
19.85	61.0	53.5	-	68.6	55.6	44.4	-	66.7
19.75	59.7	52.2	-	67.3	56.9	45.8	-	68.1
19.55	57.2	49.7	-	64.8	61.1	50.0	-	72.2
19.45	55.3	47.8	-	62.9	62.5	51.4	-	73.6
19.25	51.6	44.0	-	59.1	63.9	52.8	-	75.0
18.7	46.5	39.0	-	54.1	66.7	56.9	-	77.8
18.55	45.3	37.7	-	52.8	68.1	58.3	-	79.2
18.25	41.5	34.0	-	49.1	69.4	58.3	-	80.6
17.55	34.0	27.0	-	41.5	73.6	63.9	-	83.3
17.45	32.1	25.2	-	39.0	75.0	65.3	-	84.7
17.25	28.9	22.0	-	35.8	76.4	66.7	-	86.1
16.95	23.3	17.0	-	30.2	77.8	68.1	-	87.5
16.55	19.5	13.8	-	25.8	79.2	69.4	-	87.5
16.25	14.5	9.4	-	20.1	80.6	70.8	-	88.9
16.05	13.2	8.2	-	18.2	81.9	72.2	-	90.3
15.75	11.3	6.9	-	16.4	84.7	76.4	-	93.1
15.3	9.4	5.0	-	13.8	88.9	80.6	-	95.8
15.05	8.8	5.0	-	13.2	93.1	86.1	-	98.6
14.75	6.9	3.1	-	10.7	95.8	90.3	-	100.0
14.15	5.0	1.9	-	8.2	97.2	93.1	-	100.0
13.45	4.4	1.9	-	7.5	100.0	100.0	-	100.0

CI were obtained by 2000 stratified bootstrap. Abbreviations; CI, confidence interval.
